# Reverse Abdominoplasty: A Novel Practical Approach Using Oncoplastic Reconstruction in Managing Major Chest Wall Defects for Patients With Loco-Regional Recurrence Following Breast Cancer Surgery

**DOI:** 10.7759/cureus.19983

**Published:** 2021-11-29

**Authors:** Fiori Teklebrhan, Gheed Mahir, Stephanie Clark, Dhurka Shanthakumar, Darren K Patten, M. Z Ullah

**Affiliations:** 1 Department of Breast Surgery, Whipps Cross University Hospital - Barts Health NHS Trust, London, GBR

**Keywords:** modified radical mastectomy (mrm), aesthetic abdominoplasty, chest wall repair & reconstruction, locally advanced breast-cancer, oncological reconstruction

## Abstract

Background

Loco-regional recurrence of breast cancer in patients with large chest wall defects following mastectomy poses significant oncoplastic challenges. Reverse abdominoplasty is most commonly used to treat patients with excess upper abdominal soft tissue and laxity following massive weight loss. Widely employed as a technique for aesthetic contouring of the upper anterior trunk, as well as in augmentation mammoplasty, its use to date for reconstructive purposes is mainly limited to burns and large site surgical tumour ablation.

Method

Here we review our experience of using reverse abdominoplasty as a novel approach to filling major anterior chest wall defects in patients with cutaneous manifestations of loco-regional or distant recurrence of breast cancer.

Results

Seven patients with metastatic breast cancer underwent reverse abdominoplasty for disease recurrence following mastectomy, with good patient-reported outcomes, and minimal surgical complications. Moreover, follow-up data in the patients surveyed also showed minimal to no limitations on their activities of daily living following the procedure.

Conclusion

Here we demonstrate the successful employment of reverse abdominoplasty - a technique not usually reserved in breast oncoplastic surgery - to treat fungating breast lesions and/or other manifestations of loco-regional recurrence in metastatic breast cancer. This may herald a paradigm shift in the way surgeons approach breast cancer recurrence in patients with pre-existing major chest wall defects.

## Introduction

Breast cancer remains the leading cancer type amongst women, and the commonest cancer overall in the United Kingdom (UK). Incidence has been increasing by 19% in the UK since the 1990s, and one in eight women will be diagnosed with breast cancer during their lifetime. Approximately 7% will have metastases at the time of diagnosis [1]. Up to 18% of metastases are de novo stage IV cancer, with 82% representing relapsed disease [2].

Despite advances in medical oncology, surgery remains the mainstay in the management of invasive disease, and over 70% of patients with breast cancer will progress to surgery [3]. Surgery carries with it significant associated physical and mental morbidity, particularly after mastectomy, and thus most will be offered one of the various reconstructive techniques. Breast-conserving surgery and the various options for oncoplastic reconstruction mean that fewer patients are left with disfiguring scars and asymmetry. However, not all patients opt for or are fit for major reconstruction, and not all breast cancers are suitable for breast-conserving surgery.

Rates of locoregional recurrence following mastectomy are reported as occurring in 5-10% of patients within 10 years of primary surgical resection [4]. Patients with cutaneous manifestations of breast cancer recurrence may require surgery to improve quality of life and provide symptomatic relief from bleeding or discharging tumours, regardless of the severity of disease and long-term prognosis. Surgery for this indication (i.e. not necessarily with curative intent) can leave extensive chest wall defects, posing a substantial reconstructive challenge. Techniques used to cover such defects include autologous tissue reconstruction in the form of transverse rectus abdominis musculocutaneous flaps and fasciocutaneous flaps [5].

Reverse abdominoplasty is infrequently ascribed for reconstructive purposes. It was initially described in 1972 by Rebello and Franco who used the technique for upper abdominal wall contouring [6]. More recently, it has been used after massive weight loss surgery to reduce redundant upper abdominal tissue. Increased knowledge of the vascular supply of the anterior abdominal wall has allowed the expansion of the technique for reconstruction. We describe our experiences with seven patients who underwent reverse abdominoplasty for chest wall reconstruction after developing locoregional recurrence following resection for invasive breast cancer.

This article was previously printed as an abstract in the *European Journal of Surgical Oncology* on June 1, 2020.

## Materials and methods

Operative procedure for all cases

This study was approved at the Local Breast Surgery department. No ethical approval was required. Verbal consent was obtained by all participants for anonymised medical photography for research purposes. No human tissue was harvested.

For all seven patients that underwent reverse abdominoplasty, marking was carried out indicating the area of removal during surgery, depending on the level of cutaneous involvement over the breast tissue/chest wall. Patients had unilateral or bilateral mastectomies, dependent on extent of cutaneous metastases or patient choice. Pre-operative abdominal laxity was also estimated; all patients were also consented for probable skin grafting if adequate tissue mobilisation was not possible. However, none of these patients needed skin grafting. After mobilisation of the lower and upper flaps, we managed to close defects up to 30cm without any major tissue tension or complications.

After mastectomy, the lower flap (abdominal) was raised from cranial to caudal, below Scarpa’s fascia and anterior to the rectus sheath and external oblique aponeurosis. Initial central undermining was followed by progressive lateral undermining. Attempts were made to preserve the perforator vessels laterally but they were sacrificed if they were in the field of dissection. Dissection was carried out down to the pubic symphysis and laterally to groin creases if needed, without mobilising the umbilicus. Dissection was aided by the use of a deep or long-lighted retractor or a headlight.

Similarly, the upper flap (chest wall) after mastectomy was raised in the caudal to cranial direction, in front of pectoralis fascia and up to the level of the clavicle. Meticulous haemostasis was secured and two exudrains were used. One was kept at the lower flap and the other at the mastectomy site. The superior flap was then mobilised downwards and the inferior flap was lifted upwards and the edges were sutured at Scarpa’s fascia using 2/0 Vicryl interrupted sutures. The skin was closed in layers; deep dermal with 3/0 interrupted Vicryl stitches and skin closed with subcutaneous continuous 3/0 Monocryl sutures and steri-strips. Chest cavity exudrains were removed at 3-4 days following the procedure, and the abdominal exudrain removed after 7-10 days to allow seroma drainage.

## Results

Case 1

Patient IH, a 90-year-old female, presented with a 10cm fixed, ulcerating mass on the lateral aspect of her right breast during admission under the Orthopaedic team in 2012. She was started on anastrozole for an oestrogen receptor (ER) positive, human epidermal growth factor receptor 2 (HER2) negative grade 3 invasive ductal carcinoma (IDC). Unfortunately, she did not attend subsequent follow-up.

**Figure 1:**
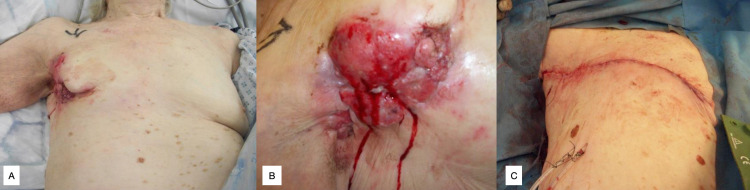
Case 1, IH Figure 1A: Preoperative appearance, ulcerating right breast mass. Figure 1B: Close up of ulcerated lesion, defect measuring 14.0x10.0cm. Figure 1C: Immediate post-operative appearance following right-sided mastectomy and reverse abdominoplasty.

IH re-presented after two years with a fungating and ulcerated right breast lesion extending into the right axilla (Figures 1A, 1B). Hormonal treatment was switched to letrozole and exemestane, however, the lesion continued to progress, severely affecting the patient’s quality of life. A multidisciplinary team (MDT) decision was made for palliative mastectomy and reverse abdominoplasty, undertaken in June 2014 (Figure 1C). Final histology confirmed a 114mm grade 3 IDC with involvement of dermis and epidermis. She was discharged on day 12 post-operatively to a rehabilitation facility. She was pleased with aesthetic outcome when later surveyed.

IH was re-admitted in August 2014 with pyrexia and confusion and sadly passed away in September 2014 from bronchopneumonia.

Case 2

Patient LT, a 54-year-old female with a history of polymyalgia rheumatica presented with a left breast grade 3 ER+, progesterone receptor (PR) positive, HER2-IDC. She underwent wide local excision (WLE) and axillary node clearance (ANC) followed by adjuvant radiotherapy and six cycles of FEC chemotherapy.

She re-presented in 2012 with right axillary node recurrence and sternal and ilial bone metastases. She was treated with palliative sternal and iliac radiotherapy and palliative chemotherapy.

**Figure 2:**
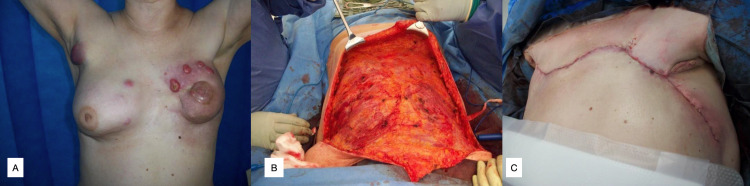
Case 2, LT Figure 2A: Left primary breast cancer (grade three invasive ductal carcinoma) with cutaneous metastases. Figure 2B: Intra-operative mobilisation of superior and inferior flaps. Figure 2C: Reverse abdominoplasty and z-plasty used to close wound.

Positron emission tomography (PET) scan in 2015 showed progression of nodal and bone disease and new adrenal disease, treated with letrozole and further radiotherapy to the axilla, supraclavicular fossa, groin nodes, and pelvic nodes. In 2017 she developed cutaneous right axillary and left breast involvement (Figure 2A).

A bilateral mastectomy with reverse abdominoplasty was performed in 2017 for regional control of multiple bilateral cutaneous metastases (Figures 2B, 2C). She was discharged on day 7 post-operatively. Wound review on day 13 showed a mostly healed wound with two small areas of dehiscence treated with minor debridement in clinic and a negative pressure wound dressing.

Final histology showed a multifocal grade 3 IDC with skin ulceration and involvement of deep margins.

LT was re-admitted in 2018 with sepsis, new bilateral pleural effusions and progression of lymphadenopathy, with para-aortic nodes causing ureteric obstruction and bilateral hydronephrosis. She sadly passed away in March 2018.

Case 3

**Figure 3:**
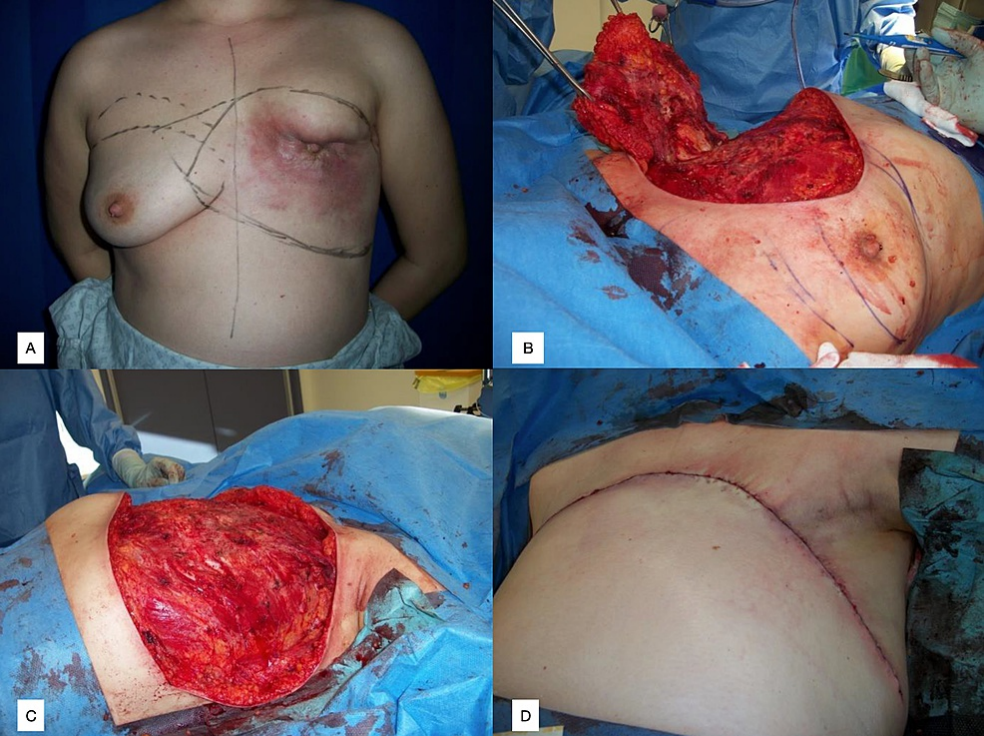
Case 3, RP Figure 3A: Anterior view of left breast lesion – grade two invasive lobular carcinoma. Defect measured 31.7cm (width), 9.6-14.7cm (superior to inferior) Figure 3B: Intra-operative view at time of removal of right breast and axillary contents. Figure 3C: Chest wall defect following bilateral mastectomy. Figure 3D: Post-operative appearance of closed abdominal and chest wall defect.

Patient RP, a 31-year-old female ex-smoker presented with left nipple inversion and was diagnosed with a T4, N1, M0, ER+, HER2- invasive lobular carcinoma (ILC). The lesion measured 90x66x85mm with pectoralis muscle invasion. She commenced neo-adjuvant FEC chemotherapy, which was changed to FEC-5 after cycle 3 following disease progression - computed tomography (CT) re-staging showed innumerable bone metastases. Due to locally advanced tumour, she was unsuitable for mastectomy and ANC in 2016 (Figure 3A). She commenced denosumab and a further six cycles of paclitaxel, goserelin and tamoxifen, completed in October 2016.

RP underwent bilateral mastectomy and reverse abdominoplasty in 2016 (Figures 3B, 3C, 3D). Final histology confirmed a post-chemotherapy grade 2 ILC, at least 120mm size, infiltrating the skin and deep margins. The right breast showed benign histology. She was discharged on day 7 post-operatively and review on day 10 showed healed surgical wounds.

RP was re-admitted in 2017 with a new malignant pleural effusion and underwent video-assisted thoracic surgery (VATS) pleural biopsy and talc pleurodesis. She was subsequently hospitalised with sepsis secondary to empyema and sadly passed away.

Case 4

Patient AF, a 55-year-old female with a history of hypertension and uterine fibroids, presented with a self-detected left breast lump. Mammogram and ultrasound (USS) showed multifocal M5 lesions. Core biopsy identified an ER+ HER2+ grade 3 IDC in all three lesions. Fine needle aspiration (FNA) for two axillary lymph nodes was positive for metastases. Staging CT showed locally advanced breast disease with skin deposits and left axillary, subpectoral, and inframammary nodal involvement. Staging MRI spine demonstrated bony metastasises in T4 and S1.

**Figure 4:**
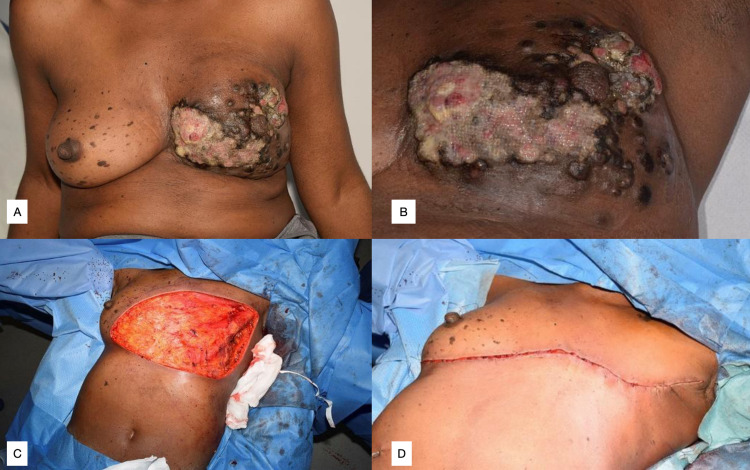
Case 4, AF Figure 4A, 4B: Anterior and close-up views of fungating lesion through the left breast with skin breakdown. Figure 4C: Anterior view of thoracic defect following left mastectomy. Figure 4D: Anterior view of closure of defect using reverse abdominoplasty.

AF commenced palliative chemotherapy with docetaxel, trastuzumab, and pertuzumab. Re-staging CT showed local disease progression with cutaneous deposits (Figures 4A, 4B) and progression of T4 bone metastases. AF subsequently underwent palliative left mastectomy with resection of chest wall lesions and reverse abdominoplasty (Figures 4C, 4D).

Final histology confirmed an 85mm grade three IDC with mixed ductal carcinoma in-situ (DCIS). AF underwent palliative chest wall, sacral and thoracic spine radiotherapy.

AF re-presented with recurrence of grade 3 IDC on the medial aspect of the mastectomy scar. Re-staging CT showed progression with new base of skull, C1, and T9 metastases. She continued radiotherapy and letrozole was commenced. AF was re-admitted with swallowing difficulties due to the progression of skull metastases and unfortunately passed away in May 2020.

Case 5

Patient MB, a 51-year-old female ex-smoker, underwent therapeutic mammoplasty in 2015 for a 19mm intermediate and high-grade DCIS with 0/3 positive nodes and margin involvement on histology. She did not undergo radiotherapy. MB declined completion mastectomy with reconstruction, instead opting to self-treat with natural remedies.

MB declined follow-up mammogram due to fear of radiation exposure, instead undergoing breast USS. Right breast USS identified a 27x15x16mm irregular mass in keeping with invasive cancer and borderline level one axillary lymph node involvement. Biopsy and FNA were declined by the patient. Four further breast clinic appointments were not attended by the patient and she was discharged from the service.

**Figure 5:**
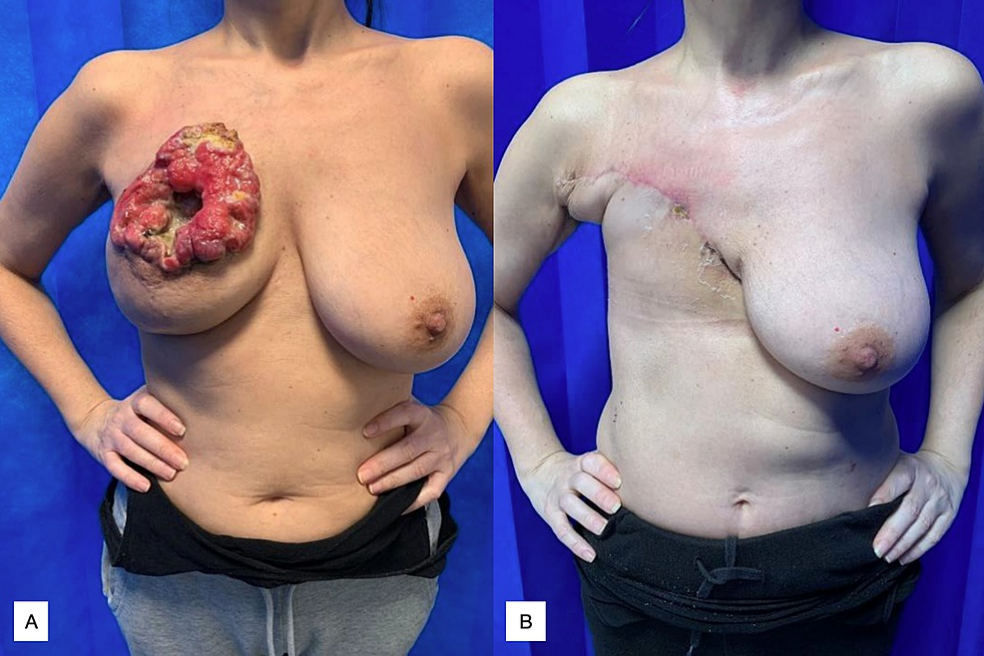
Case 5, MB Figure 5A: Regional recurrence - fungating right breast IDC on a background of ipsilateral DCIS; (IDC: invasive ductal carcinoma; DCIS: ductal carcinoma in-situ). Figure 5B: Postoperative appearance following mastectomy and reverse abdominoplasty.

MB was re-referred in 2019, with a fungating right breast mass (Figure 5A). Biopsy confirmed a poorly differentiated grade three ER+ HER2+ IDC. Re-staging CT on re-referral showed a right breast lesion infiltrating pectoralis muscle, with prominent right axillary nodes suggestive of active nodal disease. Numerous bilateral lung nodules were suspicious for pulmonary metastases. Neoadjuvant chemotherapy was declined by the patient.

MB agreed to right-sided mastectomy with reverse abdominoplasty for palliation (Figure 5B); she declined axillary clearance. Final histology confirmed over 85mm grade 3 IDC with 42mm high-grade DCIS involving the deep skeletal margins, dermis, and vascular invasion.

MB remains on tamoxifen under the care of the Oncology and Breast Surgery teams.

Case 6

Patient GM, a 48-year-old female, was treated for recurrent right breast grade 3 ER+ HER2- IDC in 2016 with skin- and nipple-sparing mastectomy, followed by ANC for one of three positive sentinel nodes. She underwent adjuvant chest wall radiotherapy and adjuvant chemotherapy. GM developed ipsilateral breast recurrence (20mm ER+ HER2- grade 3 IDC), treated with right mastectomy, adjuvant chemotherapy, and tamoxifen.

**Figure 6:**
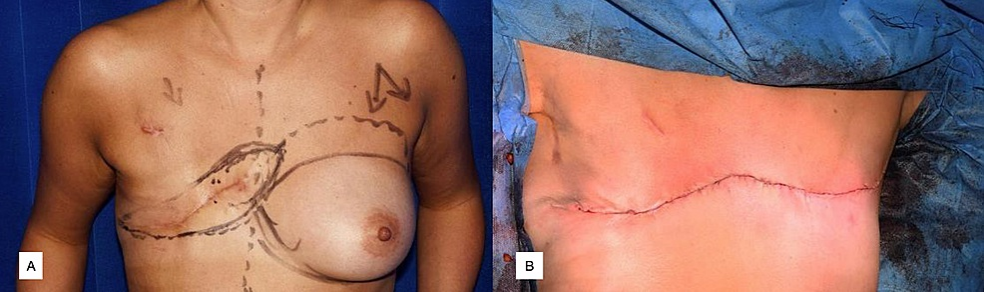
Case 6, GM Figure 6A: Preoperative appearance; recurrence at right mastectomy scar with metastatic spread to left sided axillary lymph nodes. Figure 6B: Postoperative appearance. Closure with 2'0 Vicryl interrupted deep dermal sutures & 3'0 Monocryl subcutaneous sutures.

She developed further mastectomy scar recurrence in 2020 with contralateral axillary nodal involvement (Figure 6A). She subsequently underwent left risk-reducing mastectomy with axillary node clearance and reverse abdominoplasty (Figure 6B). Histology confirmed a grade 2 ER+ HER2- IDC with left axillary nodal disease (3/12 nodes).

There were no immediate complications and GM was discharged the following day. Post-operative review identified an uncomplicated seroma requiring two aspirations in the clinic. There were no further complications.

She remains on goserelin and exemestane, under follow-up with the Breast Surgery and Oncology teams.

Case 7

Patient DV, a 46-year-old female smoker, presented with a left breast lump; histology confirmed a grade 3 IDC, with FNA-confirmed axillary metastases and sclerotic sternal metastases on staging CT and bone scan.

DV was treated with palliative chemotherapy and entered into the Efficacy and Safety of Palbociclib in Combination With Fulvestrant or Letrozole in Patients With HER2 Negative, ER+ Metastatic Breast Cancer (PARSIFAL) trial [7] in July 2017, where she was randomised to the palbociclib and letrozole arm.

She later underwent left breast radiotherapy due to enlarging cutaneous tumour progression.

**Figure 7:**
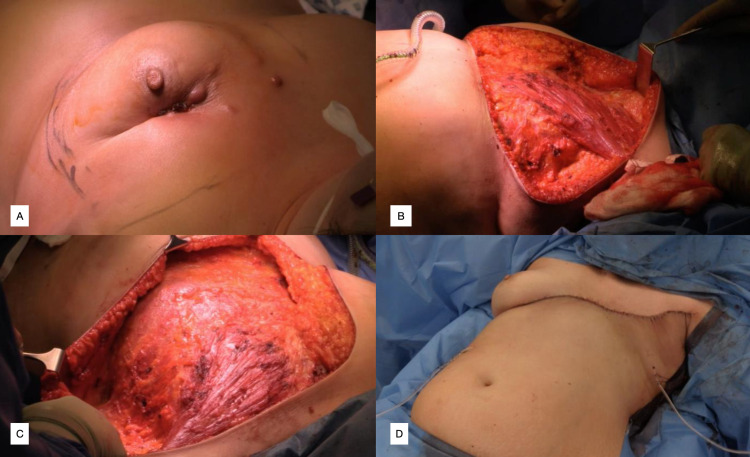
Case 7, DV Figure 7A: Preoperative appearance; left breast tumour with overlying cutaneous metastases. Figure 7B: Intra-operative appearance; chest wall defect following left mastectomy tissue removal, with superior flap raised to clavicle. Figure 7C: Intra-operative appearance; left chest wall defect following removal of breast tissue and dissection of inferior flap to level of umbilicus Figure 7D: Post-operative appearance following closure of left chest wall defect using reverse abdominoplasty technique. Two drains in situ in left axilla and anterior rectus sheath in inferior flap.

In October 2020, DV was re-referred to the breast surgery team due to the progression of breast cancer overlying the left breast skin (Figure 7A). She underwent left extended mastectomy with reverse abdominoplasty (Figures 7B, 7C, 7D). There were no immediate complications and DV was discharged home the following day. She remains on palliative chemotherapy.

Follow-up of cases

Unfortunately, of the seven patients who underwent reverse abdominoplasty, only three have survived at the time of writing: MB (case 5), GM (case 6), and DV (case 7).

Both MB and GM shared similarities in their baseline demographics and presentations (both presented in their 40s with self-detected breast mass) and underwent surgery and hormonal therapy. GM underwent more aggressive overall treatment, with additional adjuvant radiotherapy and chemotherapy.

Qualitative data on post-procedural patient-reported quality of life was obtained in the form of a Short Form-36 Health Survey Questionnaire (SF-36) [8]. The questionnaire is subdivided into categories that detail patient perception of their general health, limitations (or lack thereof) on activities, physical and emotional health problems, energy and emotions. Both MB and GM reported satisfaction with the outcome of their procedure, stating that their preoperative chest wall masses had been impacting their quality of life; both also commented on good wound healing. However, perhaps unsurprisingly, there was significant discordance in their perception of their overall health, with MB rating her general health as ‘poor’ whilst GM stated that hers was ‘very good.’ Furthermore, GM reported only mild to no limitations on all activities of daily living, and rated her health as better than it had been one year previously. She also reported no pain in the four weeks prior to answering the questionnaire, with good overall energy levels. In contrast, MB reported no limitations with activities such as climbing several flights of stairs, but struggled with more vigorous activities, citing new back pain (under investigation) as the main reason for this. DV also, interestingly, reported no limitations whatsoever on activities of daily living less than one month after surgery.

## Discussion

Large anterior trunk defects following mastectomy pose significant oncoplastic reconstructive challenges, especially in the context of persistent and/or recurrent disease. Successful aesthetic reconstruction post-mastectomy has been shown to improve a variety of emotional and functional parameters, as well as improving overall quality of life independently of prognosis [9,10]. This may be especially prudent to achieve in the palliative breast cancer setting.

Quality of life (QoL) in metastatic breast cancer is multifaceted and has been ascribed to a variety of indices such as body image, ability to perform activities of daily living, mobility and pain [11]. In one published study, up to 71% of all breast cancer patients self-reported pain to be one of the main factors impacting their QoL, with effects not just on functional capacity but overall body image [12]. Prevalence of pain also incrementally increases with disease progression; in one review 39.3% of patients reported pain after curative treatment, with rates increasing to up to 90% in advanced or metastatic disease [13,14]. For all our patients that survived to follow-up, significant improvements in pain had been reported following their reverse abdominoplasty procedure, with only mild to no limitations on activities of daily living.

Fungating chest wall tumours have also been shown to adversely affect psychosocial metrics in breast cancer patients. Complications such as pain, infection, and pruritis, as well as the cosmetic challenges posed, can be very burdensome. Chest wall resection with graft or flap reconstruction may sometimes be offered but will not always be a viable option in this patient cohort [15-17]. Here we show that the reverse abdominoplasty procedure can be used in the resection of fungating chest wall tumours following mastectomy, where more traditional reconstruction techniques are not suitable due to the size of the defects and/or underlying vascular compromise.

Reshaping and aesthetic measures normally include pedicled or free tissue flap reconstruction. However, in large, full-thickness chest wall defects, a single flap may not be able to provide adequate or complete coverage. Moreover, the high tension required to close the donor site can lead to an increased risk of local complications such as wound dehiscence and tissue deformity [18, 19]. Free flap reconstruction also relies on adequate microvascular anastomoses, the potential for which may be compromised in the event of any pre-procedural radiotherapy, and often requires longer and more complex surgery for which the patient may not be suitable.

Abdominoplasty has long been used by plastic surgeons as part of ‘body contouring’ procedures used to ‘improve the aesthetic aspect of the trunk,’ and may involve one or more surgical stages [20]. Reverse abdominoplasty is often described in conjunction with other procedures such as mammary reduction [21] and in those who have had previous lower abdominal procedures such as conventional abdominoplasty or liposuction, or those with existing infra-mammary scars [22]. Agha-Mohammadi and Hurwitz treated 88 patients with reverse abdominoplasty as part of total body contouring and reported good patient satisfaction with a low 5% complication rate [23]. Reverse abdominoplasty has also been used as an adjunct to autologous breast augmentation: Zienowicz and Karacaoglu described a novel technique, where women with a large upper abdominal pannus had de- epithelialised adipofascial flaps created that maintained their blood supply from the breast parenchyma. These flaps were passed sub-glandularly to provide autologous tissue for augmentation, and reverse abdominoplasty was used to close the defect, with the technique resulting in minimal complications [24]. Thus, its use in conjunction with mastopexy and other aesthetic breast procedures alludes to its potential in breast reconstruction.

More recently, several reconstructive uses for reverse abdominoplasty have been described. Firstly, Haik et al report the successful use of reverse abdominoplasty to treat female patients with burns involving the epigastric region and infra-mammary fold. In these three cases, the authors used tissue expanders to provide sufficient normal tissue below the area of scarring. At follow-up of one year post-surgery, all patients were satisfied with the functional and aesthetic results [25]. A handful of cases report the employment of reverse abdominoplasty for the reconstruction of central trunk defects after surgical tumour ablation. Pantelides et al successfully used reverse abdominoplasty in four patients in the reconstruction of full-thickness upper central trunk defects after oncological resection. The patient’s initial pathology ranged from recurrent breast cancer to metastatic gallbladder carcinoma at a cholecystectomy port site. They reported no cases of complete flap loss, however, one patient required revision due to marginal flap necrosis [26].

Tiong and Basiron initially described the use of reverse abdominoplasty as a stand-alone flap in the reconstruction of a large chest wall defect after bilateral mastectomy for treatment of breast cancer. Their experience with the technique was positive with the patient experiencing only minor wound dehiscence due to flap edge necrosis [27]. Similarly, di Summa et al described their experience with the use of a reverse abdominoplasty flap in a patient with local recurrence of breast cancer three months after the initial bilateral mastectomy. Reverse abdominoplasty was chosen to resurface the large anterior trunk defect as prior radiotherapy and extent of local invasion was thought to have compromised chances of adequate vascularisation and therefore achieving a good surgical outcome. In their case report, they reported no postoperative flap or donor site complications. Aesthetic outcome at an 11-month follow-up was also demonstrated to be satisfactory, and comparable to the patients treated in our breast unit [19].

## Conclusions

The provision of adequate tissue cover for large anterior trunk defects following mastectomy in patients with locoregional disease recurrence can pose significant reconstructive challenges. Commonly used techniques employed for reconstruction include pedicled or free tissue flaps, although achieving adequate tissue coverage can be difficult in large defects. Here we demonstrate that reverse abdominoplasty, a technique normally reserved for burns patients and aesthetic abdominal contouring, can instead offer an alternative method to cover massive chest wall defects in breast reconstruction for recurrent or de novo neoplasms, and avoids the need for microsurgical free tissue transfer. Our experience has shown it to be a simple and effective palliative measure in women with advanced breast disease, with large defects being covered in this way. Few postoperative complications have been demonstrated in our cohort, with concordant high clinician and patient satisfaction. Expanding the use of reverse abdominoplasty to breast oncoplastic reconstruction may therefore provide a simple and effective solution for the aesthetic challenges created by cutaneous manifestations of locoregional recurrence of breast cancer following primary surgical resection.
